# Mild Hyperbaric Oxygen Exposure Is Associated with Calculation-Method-Dependent Changes in VE/VCO_2_ During Seated Rest

**DOI:** 10.3390/biology15151254

**Published:** 2026-07-30

**Authors:** Takehira Nakao, Toru Hirata, Atsushi Saito

**Affiliations:** 1 Faculty of Human Sciences, Kyushu Sangyo University, 3-1 Matsukadai 2, Higashi-ku, Fukuoka 813-8503, Japan; 2 Faculty of Medicine, Miyazaki University, 5200 Kihara, Kiyotake-cho, Miyazaki 889-1692, Japan; 3 Faculty of Human-Environment Studies, Kyushu University, 744 Motooka, Nishi-ku, Fukuoka 819-0395, Japan

**Keywords:** hyperbaric oxygen, partial pressure of inspired oxygen, ambient pressure, oxygen concentration, ventilatory response, gas exchange

## Abstract

Mild hyperbaric oxygen (HBO) exposure might influence ventilatory and gas-exchange responses. We investigated the effects of mild HBO exposure at 1.3 atmospheres absolute (ATA) on VE/VCO_2_ and related variables at rest in healthy young men. Using a single-group repeated-measures design, pre, steady (20–50 min at 1.3 ATA), and post-phases were compared in 16 participants. The primary outcome was VE/VCO_2_, calculated as minute ventilation (VE) divided by carbon dioxide output (VCO_2_); VE, VCO_2_, and partial pressure of end-tidal CO_2_ (PETCO_2_) were secondary outcomes. Linear mixed models and Bonferroni-adjusted pairwise comparisons were used. Using the primary bin-wise calculation, VE/VCO_2_ increased significantly from 39.9 ± 1.0 in the pre-phase to 43.8 ± 1.0 in the steady phase and decreased to 38.8 ± 1.0 in the post-phase. VE and PETCO_2_ showed no phase effects; VCO_2_ decreased significantly during the post-phase. However, an alternative calculation based on phase-averaged VE divided by phase-averaged VCO_2_ produced the opposite phase-dependent pattern. Among eight participants completing both HBO and normobaric normoxic conditions, the exploratory Condition × Phase interaction was significant for both calculations, although phase patterns differed between methods. Thus, the direction of the VE/VCO_2_ change depended on the calculation method, and its physiological significance remains uncertain.

## Simple Summary

It is not fully understood how respiratory patterns at rest change in an environment with slightly higher atmospheric pressure and oxygen concentration, despite the increased supply of oxygen to the body. In this study, we investigated changes in respiration and carbon dioxide output in 16 healthy young men whilst they were sitting at rest in such an environment. Using the primary calculation method, the minute ventilation-to-carbon dioxide output ratio increased slightly during exposure and fell afterwards, without clear changes in minute ventilation or carbon dioxide indicators in exhaled breath. However, an alternative calculation method produced the opposite pattern. Thus, the direction of change depended on how the ratio was calculated, and its physiological significance remains uncertain. These findings provide a basis for future studies of respiratory responses to mild hyperbaric oxygen exposure.

## 1. Introduction

Hyperbaric oxygen (HBO) exposure increases the partial pressure of inspired oxygen through elevated ambient pressure and oxygen concentration, thereby enhancing oxygen delivery to the blood and tissues. Clinically, this principle has been applied in the development of hyperbaric oxygen therapy (HBOT) for treating conditions, such as decompression sickness, wound healing, ischaemic diseases, and infectious diseases. To date, numerous reports have been published on the clinical applications, mechanisms of action, safety, adverse events, and future research directions of HBOT [1,2,3]. However, HBO is not merely a means to increase oxygen supply but is a complex physiological stimulus that involves circulation, ventilation, metabolic regulation, and the autonomic nervous system.

A systematic review and meta-analysis on acute hyperoxia exposure has shown that normobaric hyperoxia exposure may reduce cardiac output and increase systemic vascular resistance [4]. Furthermore, an HBO environment has been classified as a special environment alongside microgravity and low-pressure, hypoxic environments; moreover, despite its therapeutic potential through increasing oxygen supply, it poses risks, such as oxygen toxicity and oxidative stress [5]. Consequently, it is necessary to gain a better understanding of the physiological response to HBO exposure by performing an integrated assessment that encompasses not only increased oxygen supply but also circulation, ventilation, and metabolic regulation.

In recent years, mild HBO environments, which involve lower pressure conditions than conventional medical HBOT, have attracted attention in fields such as health sciences, environmental physiology, and human biology. Mild HBO is generally applied at a lower pressure than medical HBOT, typically around 1266–1317 hPa, and can provide an oxygen partial pressure equivalent to approximately 35–40% oxygen under normobaric conditions. Its potential applications have been reported in areas such as improving skeletal muscle oxidative metabolic capacity, treating metabolic diseases, and creating models of neurodegenerative diseases [6]. Furthermore, human studies on mild HBO have reported that it exerts effects on autonomic nervous activity, immune response, and oxidative stress [7]. However, while studies on mild HBO environments have examined circulatory and metabolic responses, skeletal muscle function, potential applications in the nervous system, and effects on fatigue and recovery [6,7,8], ventilatory and gas-exchange responses at rest in healthy adults—particularly the ventilatory response to CO_2_ production—have not been sufficiently investigated.

Hyperoxia may also influence respiratory regulation and CO_2_ dynamics. Studies on healthy individuals have shown that inhaling 30% and 75% oxygen stimulates ventilatory responses, suggesting that the Haldane effect is the primary candidate mechanism for hyperoxic hyperventilation [9]. Furthermore, antioxidant interventions have been reported to reduce reactive oxygen species (ROS) production, ROS accumulation, and increased ventilation during hyperoxic exposure, suggesting that ROS are involved in the regulation of the hyperoxic hyperventilation response [10]. Furthermore, basic research using rats has revealed that hyperbaric oxygen and oxidative stimuli stimulate CO_2_/H^+^-sensitive neurons in the brainstem [11], and the involvement of O_2_/CO_2_-sensitive neurons in the medulla oblongata has also been suggested as a background factor that plays a role in hyperoxic hyperventilation and central nervous system oxygen toxicity [12]. Therefore, to understand respiratory responses during HBO exposure, it is important to evaluate not only minute ventilation but also CO_2_ production, CO_2_ output, and indices of CO_2_ homeostasis.

The VE/VCO_2_ ratio, calculated by dividing minute ventilation (VE) by carbon dioxide output (VCO_2_), is considered a useful indicator that reflects the ventilatory response to metabolic CO_2_ production. For example, VE/VCO_2_ has been reported to be a comprehensive indicator of ventilatory efficiency that is influenced by factors such as ventilatory volume, CO_2_ production, alveolar ventilation, dead-space ventilation, arterial CO_2_ partial pressure, and the ventilatory–haemodynamic relationship [13]. Therefore, we considered that evaluating VE/VCO_2_ during HBO exposure would allow us to examine changes in the ratio of ventilation to metabolic CO_2_ output, which cannot be assessed from changes in ventilation alone. Furthermore, by simultaneously evaluating the partial pressure of end-tidal CO_2_ (PETCO_2_) as a supplementary measure, it would be possible to interpret whether changes in VE/VCO_2_ are accompanied by clear CO_2_ retention or depletion.

However, few studies have examined the effects of mild HBO exposure at approximately 1.3 atmospheres absolute (ATA) on resting VE/VCO_2_, VE, VCO_2_, and PETCO_2_ in healthy young adults, focusing on the steady-state exposure phase. In particular, it is unclear whether mild HBO exposure alters VE itself or changes the relative amount of ventilation in response to CO_2_ production.

Therefore, in this study, we aimed to investigate the effects of mild HBO exposure at 1.3 ATA on ventilatory and gas-exchange responses at rest in healthy young men. The primary outcome measure was VE/VCO_2_, and VE, VCO_2_, and PETCO_2_ were also assessed. We hypothesised that mild HBO exposure would transiently increase VE/VCO_2_ without a clear increase in minute ventilation itself.

## 2. Materials and Methods

### 2.1. Study Design

This environmental physiology study investigated the effects of transient exposure to a mild hyperbaric hyperoxic environment on ventilatory and gas-exchange responses at rest in healthy young men. The study was designed as a single-group repeated-measures analysis that compared three phases in the same participants: before, during, and after hyperbaric hyperoxic exposure. These phases were defined as the ‘pre-phase’ (resting period under normal atmospheric pressure before pressurisation), ‘steady phase’ (steady-state exposure period after reaching 1.3 ATA), and ‘post-phase’ (resting period under normal atmospheric pressure after decompression).

**Figure 1:**
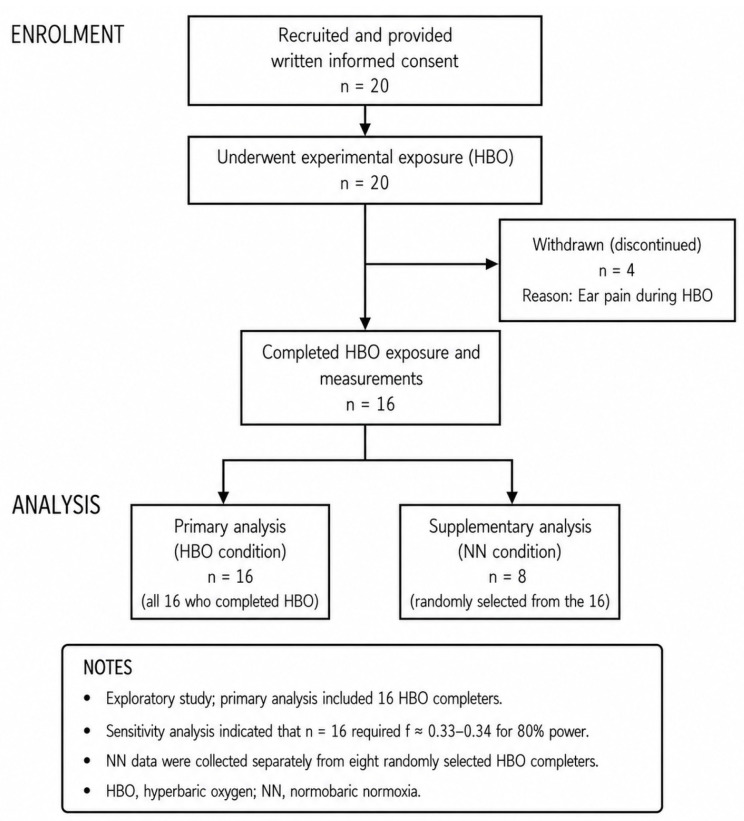
Flow diagram of participant enrolment and analysis. Twenty participants were recruited, and 16 completed the HBO condition and measurements. The primary analysis included all 16 participants who completed the HBO condition, whereas the supplementary NN analysis included 8 participants who were randomly selected from among the HBO completers. HBO, hyperbaric oxygen; NN, normobaric normoxia.

The main analysis included 16 participants who completed the HBO condition (Figure 1). Furthermore, to explore whether similar phase-dependent changes occurred during seated rest without pressurisation or oxygen enrichment, resting measurements were conducted under normobaric normoxic (NN) conditions in a subset of participants who had completed the HBO protocol. Data from the NN condition were not included in the main analysis but were used as supplementary reference data.

This study was not conducted for medical purposes but to elucidate ventilatory and gas-exchange responses at rest in a mild HBO environment. It was conducted in accordance with the principles of the Declaration of Helsinki and was approved by the Ethics Committee. Written informed consent was obtained from all participants. All participants were fully informed of the study objectives, methods, anticipated burdens, and risks and provided written informed consent.

### 2.2. Study Participants

Participants were recruited between 17 November 2025 and 30 March 2026 via university noticeboards, online announcements, and social media posts. Twenty healthy young men were provided with a full explanation of the study’s objectives, experimental procedures, and anticipated burdens and risks, and their written consent was obtained.

The inclusion criteria were as follows: no history of cardiovascular, respiratory, or metabolic diseases; no smoking habits; and no contraindications to exposure to a hyperbaric environment. The exclusion criteria were as follows: under 20 years of age, a history of obvious medical conditions, smoking habits, difficulty with ear equalisation or other factors making adaptation to a hyperbaric environment difficult, or participation deemed inappropriate by the principal investigator.

Four participants reported experiencing ear pain during HBO exposure and discontinued the measurements; therefore, 16 participants completed the HBO condition and were included in the primary analysis. Furthermore, to explore the effects of the passage of time and seated rest itself in supplementary analyses, eight of the 16 participants who completed the HBO condition were selected using a computer-based random selection procedure, and similar resting measurements were conducted under NN conditions. These NN measurements were conducted on a separate day from the HBO measurements. The NN subset was descriptively reviewed and showed no apparent deviation in age or anthropometric characteristics from the full HBO cohort.

Because this was an exploratory environmental physiology study and given the feasibility of the experiment, the main analysis included the 16 participants who completed the HBO condition. A statistical power sensitivity analysis based on a one-way repeated-measures analysis of variance indicated that an effect size of approximately f = 0.33–0.34 was required to achieve approximately 80% power with *n* = 16. Therefore, to interpret the results, we evaluated the estimated values, 95% confidence intervals, and effect sizes in addition to statistical significance.

### 2.3. Experimental Conditions and Measurement Parameters

#### 2.3.1. Experimental Conditions and Exposure Protocol

The following conditions were maintained in the HBO environment: an absolute pressure of 1.31 ± 0.01 ATA, a chamber oxygen concentration of 27.2–27.6%, a temperature of 24.8 ± 0.7 °C, and a humidity of 57.8 ± 6.7%. This chamber oxygen concentration at 1.31 ± 0.01 ATA corresponded to a normobaric-equivalent oxygen fraction of approximately 35–36%. The following conditions were maintained in the NN environment: an absolute pressure of 1.00 ± 0.01 ATA, an oxygen concentration of 20.9% (approximately 21%), a temperature of 25.4 ± 1.3 °C, and a humidity of 39.5 ± 3.1%.

The HBO environment was constructed using an artificial environmental control chamber (Japan Pressure Bulk Industries Co., Ltd., Shizuoka, Japan), and the pressurisation and depressurisation rates were set at approximately 0.07 ATA/min. The NN environment was set up in a standard laboratory that was equipped with similar measurement facilities. The hyperbaric chamber that was used complied with domestic operational safety standards (mild HBO Type II, ≤1.4 ATA) and was distinct from medical HBOT equipment.

All measurements were conducted individually, with one examiner and one participant. Experiments were scheduled in the morning whenever possible; however, the exact time of day was not fully standardised. Participants were instructed to avoid strenuous physical activity on the day of testing, and measurements were generally performed between sedentary classroom sessions. Meal timing, caffeine intake, sleep status, psychological stress, and circadian influences were not strictly controlled.

Under the HBO condition, participants remained seated and at rest inside the chamber. Sleeping and physical activity were prohibited during the measurement; only reading, listening to music, and the use of smartphones were permitted. Following a 3 min resting measurement at normal atmospheric pressure, the chamber was pressurised to 1.3 ATA over approximately 12 min, and when the absolute pressure reached 1.3 ATA, the participant was subjected to 60 min of HBO exposure. Subsequently, the pressure was reduced to atmospheric pressure over approximately 9 min, which was followed by a 3 min resting measurement at atmospheric pressure after decompression.

The primary analysis interval was set at 20–50 min after reaching the target pressure to avoid transient fluctuations after the absolute pressure reached 1.3 ATA and to ensure a stable steady state. The pressure during this interval was 987.5 ± 3.6 mmHg, which is equivalent to approximately 1.3 ATA. The HBO condition was not intended to simulate medical HBOT but was designed as an environmental physiological condition to evaluate ventilatory and gas-exchange responses at rest in a mildly hyperbaric, hyperoxic environment.

Under the NN condition, which was used to obtain data for the supplementary analyses, participants remained seated at rest in a normobaric normoxic environment, and exhaled gas measurements were taken during time periods corresponding to each measurement phase under the HBO condition. The NN condition was implemented as a supplementary reference condition to explore whether similar phase-dependent changes occurred during seated rest without pressurisation or oxygen enrichment and was not included in the main analysis.

#### 2.3.2. Measurement of Physical Characteristics

Before the experiment, the physical characteristics of the participants were assessed. Height was measured using a standard height rod (A&D Co., Ltd., Tokyo, Japan). Body weight, body fat mass, body fat percentage, total body water, and basal metabolic rate were assessed using a bioelectrical impedance analyser (InBody 770; InBody Japan Co., Ltd., Tokyo, Japan). Body mass index (BMI) was calculated by dividing body weight by the square of height.

The physical characteristics of the 16 participants who were included in the main analysis are shown in Table 1. The participants’ mean age was 22.1 ± 3.1 years, mean height was 173.5 ± 6.9 cm, body weight was 73.5 ± 10.8 kg, BMI was 24.3 ± 3.0 kg·m^−2^, body fat mass was 13.1 ± 6.5 kg, body fat percentage was 17.3 ± 6.3%, total body water was 44.2 ± 5.4 L, and basal metabolic rate was 1674 ± 159 kcal·day^−1^.

#### 2.3.3. Breath Gas Analysis

Exhaled gas parameters were measured continuously on a breath-by-breath basis using a mass spectrometer for bio-gas analysis (ARCO-2000, ARCO SYSTEM Inc., Chiba, Japan). Subjects wore a mask for exhaled gas measurement, and exhaled gases were analysed via a sampling tube. The measured parameters were VE, VO_2_, VCO_2_, respiratory rate (RR), tidal volume (TVE), partial pressure of end-tidal oxygen (PETO_2_), and PETCO_2_. Heart rate (HR) was detected using a Polar H10 heart rate transmitter (Polar Electro Oy, Kempele, Finland) and synchronously input to the ARCO-2000 via a Polar Receiver PR-10, where it was recorded simultaneously with the respiratory gas parameters. VE/VCO_2_ was calculated by dividing VE by VCO_2_ after both variables had been expressed in consistent volume units. Furthermore, the respiratory exchange ratio (R) was calculated by dividing VCO_2_ by VO_2_. Before each measurement, the gas analyser was calibrated under normobaric conditions using ambient air and a certified calibration gas. Although no independent validation experiment was performed under hyperbaric conditions, the manufacturer confirmed that the analyser could be operated continuously and measure gas concentrations at pressures up to 1.3 ATA. The built-in barometer had a measurement range of 350–1000 mmHg, and all recorded chamber pressures remained below 1000 mmHg. VE, VO_2_, and VCO_2_ were converted from ATPS to BTPS using the chamber ambient pressure measured by the analyser’s built-in barometer. PETCO_2_ was expressed as partial pressure in mmHg and was therefore not subject to BTPS volume correction.

#### 2.3.4. Processing of Exhaled Gas Data

The exhaled gas data obtained using the breath-by-breath method were averaged every 30 s and organised by bin. The analysis intervals were defined as follows: the pre-phase, comprising the resting period at normal atmospheric pressure before pressurisation; the steady phase, comprising the steady-state exposure period of 20–50 min after the absolute pressure reached 1.3 ATA; and the post-phase, comprising the resting period at normal atmospheric pressure after depressurisation.

For each 30 s bin, VE/VCO_2_ was calculated by dividing the bin-averaged VE by the bin-averaged VCO_2_ after both variables had been expressed in consistent volume units. Before phase averaging, measurement errors and physiologically implausible values were identified. Values were excluded when VE/VCO_2_ was >55, VE/VO_2_ was ≥60, PETCO_2_ was <25 or >50 mmHg, R was <0.70 or >1.20, or VO_2_ or VCO_2_ was ≤0. Considering the possibility of physiological variations under hyperbaric conditions, values for which VE/VCO_2_ fell within the range of 50–55 were not excluded, provided that they were not accompanied by obvious abnormalities in other respiratory gas parameters. After these exclusions, VE/VCO_2_ and the other variables were averaged within each phase for each participant before statistical analysis.

#### 2.3.5. Statistical Analysis

IBM SPSS Statistics ver. 27 (IBM Corp., Armonk, NY, USA) was used for statistical analysis. The primary analysis included the 16 participants who completed the HBO condition. The primary outcome measure was VE/VCO_2_, and the secondary outcome measures were VE, VCO_2_, and PETCO_2_. A linear mixed model (LMM) was used to assess the changes in each outcome measure across the three phases (pre, steady, and post).

In the LMM, the phase (pre, steady, and post) was treated as a fixed effect, and participant ID was treated as the unit of repeated measurement. The restricted maximum likelihood method was used for estimation, and an autoregressive covariance structure (AR(1)) was specified for the repeated measures. After evaluating the main effect of phase, pairwise comparisons between pre–steady, steady–post, and pre–post were performed using Bonferroni correction.

Results were presented as estimated marginal means (EMM) ± standard error (SE) and 95% confidence intervals (CI). Furthermore, to assess the magnitude of change between phases, Hedges’ *g*_av_ was calculated as an effect size. The statistical significance level was set at *p* < 0.05 for two-tailed tests.

To assess the sensitivity of the VE/VCO_2_ findings to the calculation method, a sensitivity analysis was conducted in which VE/VCO_2_ was recalculated for each participant and phase as phase-averaged VE divided by phase-averaged VCO_2_. The recalculated values were analysed using the same LMM specification and Bonferroni-adjusted pairwise comparisons as in the primary analysis, and Hedges’ *g*_av_ was calculated using the same method.

As an exploratory supplementary analysis, a similar LMM analysis was conducted under normobaric normoxic conditions in eight of the 16 participants who completed the HBO condition. The HBO and NN conditions were first analysed separately. In addition, exploratory Condition × Phase interaction analyses were conducted in the eight participants who completed both conditions. Condition, Phase, and the Condition × Phase interaction were included as fixed effects. Participant ID was included as a random intercept, and Phase was specified as a repeated measure within participant and condition using an AR(1) covariance structure. When a significant interaction was detected, simple effects of Phase within each condition and Bonferroni-adjusted pairwise comparisons were examined. These interaction analyses were conducted for both the primary and alternative VE/VCO_2_ calculations and were considered exploratory because the NN condition involved only eight participants, was conducted on a separate day, and was not part of a fully randomised and counterbalanced crossover design. No baseline scaling, normalisation, or baseline adjustment based on the NN condition was applied to the primary HBO linear mixed model.

## 3. Results

### 3.1. Changes in Barometric Pressure and Minute Ventilation over Time

**Figure 2:**
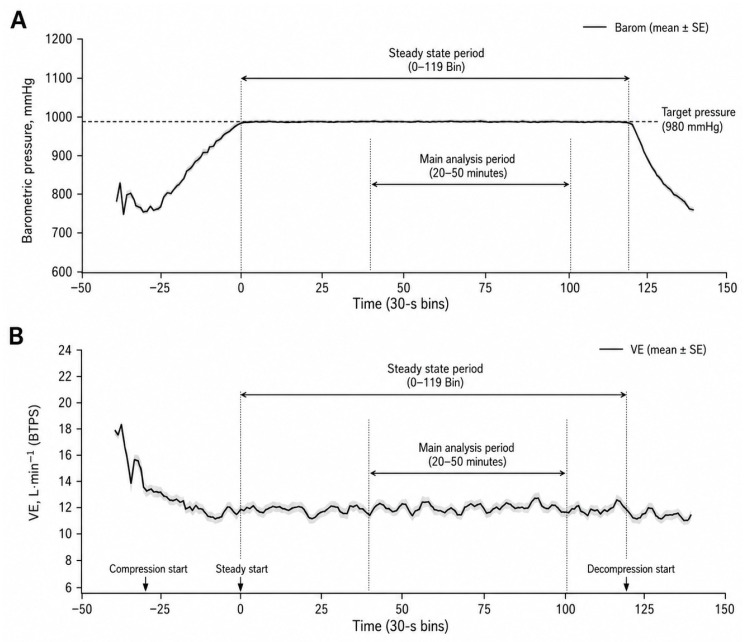
Time courses of (**A**) measured ambient barometric pressure (Barom) and (**B**) minute ventilation (VE) during mild hyperbaric oxygen exposure. Values in both panels are presented as mean ± SE for 16 participants, with grey bands indicating SE. Barom was recorded using the gas analyser’s built-in barometer, and no additional smoothing was applied. The SE band for Barom is narrow because between-participant variability in measured ambient pressure was minimal. The horizontal dashed line in panel A indicates the target pressure of approximately 980 mmHg. Vertical dotted lines and arrows indicate compression start, attainment of steady state, the start and end of the main analysis period, and decompression start. One bin represents 30 s. Barom, barometric pressure; VE, minute ventilation; BTPS, body temperature and pressure, saturated.

Figure 2 shows the changes in barometric pressure (Barom) and VE over time during high-pressure exposure. Barom rose during the pressurisation phase and was maintained at 987.5 ± 3.6 mmHg during the main analysis period, corresponding to 20–50 min after the start of the steady phase. Although there were transient fluctuations in VE before and during pressurisation, it remained relatively stable throughout the steady phase. In other words, stable high-pressure conditions were maintained during the main analysis period, and no clear gradual increase in VE was observed during exposure.

### 3.2. Key Physiological Responses During the Steady-State Exposure Interval

Table 2 shows the key physiological responses during the primary analysis interval in the 1.3 ATA steady phase. During the main analysis interval, corresponding to 20–50 min after the steady phase started, VE was 11.9 ± 1.8 L·min^−1^ (95% CI: 10.9–12.8), VCO_2_ was 298.8 ± 49.9 mL·min^−1^ (95% CI: 272.2–325.4), VE/VCO_2_ was 43.8 ± 4.1 (95% CI: 41.6–46.0), and PETCO_2_ was 34.9 ± 2.8 mmHg (95% CI: 33.4–36.4).

### 3.3. Phase-Dependent Changes in Ventilatory and Gas-Exchange Variables

**Figure 3:**
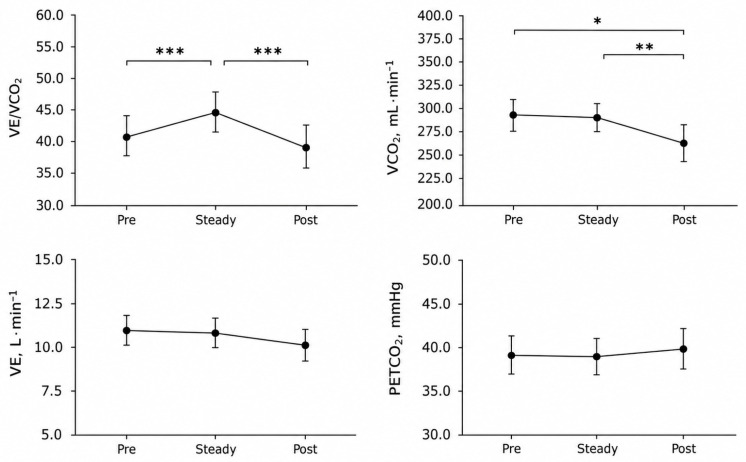
Phase-dependent changes in ventilatory and gas-exchange responses during mild hyperbaric oxygen exposure. VE/VCO_2_ values were based on the primary calculation, in which bin-wise ratios were averaged within each phase. Values are presented as estimated marginal means (EMM) ± standard error (SE) for the 16 participants. Pairwise comparisons were based on the linear mixed model analysis shown in Table 3 and were Bonferroni-adjusted. Table 3 presents EMM ± SE with 95% confidence intervals. * *p* < 0.05, ** *p* < 0.01, *** *p* < 0.001.

Figure 3 shows the phase-dependent changes in ventilatory and gas-exchange variables during hyperbaric exposure. VE/VCO_2_ increased significantly from the pre-phase to the steady phase and decreased significantly from the steady phase to the post-phase (both *p* < 0.001). By contrast, no significant differences in VE were observed between phases. VCO_2_ was significantly lower in the post-phase than in the pre- and steady phases (pre–post: *p* = 0.020, steady–post: *p* = 0.009). No significant differences in PETCO_2_ were observed between phases.

### 3.4. Linear Mixed-Effects Model Analysis of Phase-Dependent Changes in Ventilatory and Gas-Exchange Variables

Table 3 presents the results of the linear mixed-effects model analysis of phase-dependent changes in ventilatory and gas-exchange variables. A significant phase effect was observed for VE/VCO_2_ (F = 16.984, *p* < 0.001). The EMM of VE/VCO_2_ increased significantly from 39.9 ± 1.0 (95% CI: 37.8–42.0) in the pre-phase to 43.8 ± 1.0 (95% CI: 41.7–45.9) in the steady phase (*p* < 0.001) and subsequently decreased significantly to 38.8 ± 1.0 (95% CI: 36.7–40.9) in the post-phase (*p* < 0.001). However, no significant difference was observed between the pre- and post-phases (*p* = 0.455).

No significant phase effect was observed for VE (F = 2.426, *p* = 0.105), and no significant differences were found in pairwise comparisons between the pre-, steady, and post-phases.

A significant phase effect was observed for VCO_2_ (F = 8.009, *p* = 0.002). Although VCO_2_ was not significantly different between the pre- and steady phases, it decreased significantly from 302.0 ± 13.1 (95% CI: 274.6–329.3) in the pre-phase and 298.8 ± 13.1 (95% CI: 271.5–326.1) to 274.6 ± 13.1 (95% CI: 247.3–301.9) in the post-phase (Steady–Post; *p* = 0.009, Pre–Post; *p* = 0.020).

No significant phase effect was observed for PETCO_2_ (F = 1.371, *p* = 0.281), and no significant differences were found in any pairwise comparisons.

### 3.5. Sensitivity Analysis of the VE/VCO_2_ Calculation

To assess the sensitivity of the VE/VCO_2_ findings to the calculation method, a sensitivity analysis was conducted in which VE/VCO_2_ was recalculated for each participant and phase as phase-averaged VE divided by phase-averaged VCO_2_. A significant phase effect was observed (*F* = 12.313, *p* < 0.001). The estimated marginal means were 42.5 ± 1.2 in the pre-phase, 39.9 ± 1.2 in the steady phase, and 43.2 ± 1.2 in the post-phase. Bonferroni-adjusted pairwise comparisons showed that VE/VCO_2_ was significantly lower in the steady phase than in the pre-phase (pre–steady mean difference, 2.55; 95% CI, 0.54–4.56; *p* = 0.010) and post-phase (steady–post mean difference, −3.24; 95% CI, −5.25 to −1.23; *p* < 0.001), whereas no significant difference was observed between the pre- and post-phases (*p* = 1.000). The phase-dependent pattern was opposite to that observed in the primary analysis (Table S3).

### 3.6. Exploratory Condition × Phase Interaction Analyses of VE/VCO_2_

Among the eight participants who completed both conditions, the primary calculation, in which bin-wise VE/VCO_2_ ratios were averaged within each phase, showed a significant Condition × Phase interaction (*F* = 10.577, *p* < 0.001). The simple effect of Phase was significant within the HBO condition (*F* = 22.126, *p* < 0.001) but not within the NN condition (*F* = 1.474, *p* = 0.248). Under HBO, VE/VCO_2_ increased significantly from the pre-phase to the steady phase and decreased significantly from the steady phase to the post-phase, whereas no Bonferroni-adjusted pairwise phase differences were detected under NN conditions.

For the alternative calculation, in which VE/VCO_2_ was calculated as phase-averaged VE divided by phase-averaged VCO_2_, the Condition × Phase interaction was also significant (*F* = 5.576, *p* = 0.010). The simple effect of Phase was significant within both the HBO (*F* = 6.826, *p* = 0.004) and NN conditions (*F* = 3.404, *p* = 0.049). However, after Bonferroni adjustment, only the steady–post comparison under HBO was significant (*p* = 0.003), and no pairwise comparison under NN conditions reached *p* < 0.05. Thus, although a Condition × Phase interaction was detected using both calculation methods, the within-condition phase pattern and specific pairwise contrasts depended on the VE/VCO_2_ calculation method (Table S4).

### 3.7. Supplementary Data on Steady-State Exposure Characteristics and Physiological Responses During 20–50 min of Exposure Under 1.3 ATA Conditions

Supplementary Table S1 presents the exposure conditions and supplementary physiological responses during the 20–50 min primary analysis interval at 1.3 ATA. Barom was maintained at 987.5 ± 3.6 mmHg [95% CI: 985.7–989.4], and the primary analysis interval occurred under stable hyperbaric conditions. During this period, RR was 17.9 ± 3.2 breaths·min^−1^ [95% CI: 16.2–19.6], TVE was 0.77 ± 0.22 L [95% CI: 0.66–0.89], VO_2_ was 349.2 ± 59.6 mL·min^−1^ [95% CI: 317.4–380.9], and R was 0.86 ± 0.05 [95% CI: 0.83–0.89]. Furthermore, VE/VO_2_, PETO_2_, and HR were 38.9 ± 3.9 [95% CI: 36.8–41.0], 179.1 ± 5.0 mmHg [95% CI: 176.4–181.8], and 68.5 ± 7.9 beats·min^−1^ [95% CI: 64.3–72.7], respectively.

### 3.8. Supplementary Data Under NN Conditions

Supplementary Table S2 presents the results of LMM analyses under normal pressure and normal oxygen conditions in the supplementary cohort (*n* = 8). Under NN conditions, no significant phase effect was observed for VE/VCO_2_ (F = 1.94, *p* = 0.181). The EMMs were 35.8 ± 1.8 [95% CI: 31.8–39.7] for the pre-phase, 34.3 ± 1.8 [95% CI: 30.4–38.3] for the steady phase, and 33.4 ± 1.8 [95% CI: 29.5–37.4] for the post-phase. No significant phase effects were observed for VCO_2_ or PETCO_2_ (F = 1.06, *p* = 0.373 and F = 1.94, *p* = 0.183, respectively). By contrast, a significant overall phase effect was observed for VE (F = 5.61, *p* = 0.016); however, none of the Bonferroni-adjusted pairwise comparisons reached statistical significance (Pre–Steady: *p* = 0.115; Steady–Post: *p* = 1.000; Pre–Post: *p* = 0.055).

## 4. Discussion

In this study, we investigated the effects of mild HBO exposure at 1.3 ATA on ventilatory and gas-exchange responses in healthy young men during seated rest. In the primary analysis, VE/VCO_2_ increased significantly during the steady phase, whereas VE, VCO_2_, and PETCO_2_ did not change significantly during this phase. During the post-phase, VCO_2_ decreased significantly compared with both the pre- and steady phases, and VE/VCO_2_ decreased from the steady phase to the post-phase, whereas PETCO_2_ remained unchanged. Thus, the primary analysis indicated a modest, transient increase in VE/VCO_2_ during mild HBO exposure, without significant changes in VE or PETCO_2_.

However, the sensitivity analysis using phase-averaged VE divided by phase-averaged VCO_2_ showed the opposite phase-dependent pattern. Accordingly, the direction of the VE/VCO_2_ response depended on the calculation method, and the primary directional finding should be interpreted cautiously.

Previous studies on mild HBO environments have examined circulation, metabolic responses, skeletal muscle function, potential applications in neurological disease models, and fatigue and recovery responses. However, ventilatory and gas-exchange responses at rest in healthy adults, particularly the relationship between ventilation and CO_2_ output, have not been sufficiently investigated [6,7,8]. The present study addressed this gap and further showed that the observed phase-dependent VE/VCO_2_ pattern was sensitive to the calculation method.

### 4.1. Physiological Interpretation of the VE/VCO_2_ Increase in the Primary Analysis

VE/VCO_2_ is an index obtained by dividing minute ventilation by CO_2_ output and reflects the extent to which ventilation occurs in response to metabolic CO_2_ production. In the fields of exercise physiology and clinical exercise testing, the relationship between VE and VCO_2_ is used to assess ventilation efficiency, and an increase in VE/VCO_2_ is interpreted as an indicator of a state requiring comparatively high ventilation relative to CO_2_ output. Furthermore, VE/VCO_2_ has been reported to be a composite indicator influenced by factors such as ventilation volume, CO_2_ production, alveolar ventilation, dead-space ventilation, arterial blood CO_2_ partial pressure, and the ventilation–perfusion ratio [13,14]. However, this study did not involve an exercise stress test but focused on mild HBO exposure during seated rest. Therefore, the increase in VE/VCO_2_ observed in the primary analysis cannot be interpreted as being synonymous with a clinical reduction in ventilatory efficiency. Rather, the primary-analysis increase in VE/VCO_2_ should be interpreted as a modest, transient increase in the ratio of total ventilation to metabolic CO_2_ output. Based on the primary-analysis estimated marginal means, VE/VCO_2_ increased from 39.9 in the pre-phase to 43.8 in the steady phase, corresponding to an increase of 3.9, or approximately 9.8%, with an absolute Hedges’ *g*_av_ of 0.97. Although the standardised effect size was substantial, its biological and clinical significance remains uncertain because VE and PETCO_2_ were unchanged, relevant physiological determinants were not assessed, and the direction of change was not robust to the calculation method.

### 4.2. Potential Explanations for the VE/VCO_2_ Increase in the Primary Analysis

The mechanism underlying the transient increase observed in the primary VE/VCO_2_ analysis cannot be determined from the present data. Previous studies have shown that inhalation of 30% and 75% oxygen can stimulate ventilation and have suggested that the Haldane effect may contribute to this response [9]. ROS-related signalling has also been suggested to contribute to ventilatory responses during hyperoxic exposure [10]. However, the exposure conditions used in these studies differed from the mild HBO condition used in the present study, and VE did not increase significantly during the steady phase. Therefore, the present findings do not directly demonstrate that hyperoxic hyperventilation occurred.

One possible explanation is the Haldane effect. If haemoglobin oxygenation increased during mild HBO exposure, changes in blood CO_2_ binding and release may have contributed to the observed increase in VE/VCO_2_ [9,15]. Another possible explanation is modulation of central respiratory regulation through ROS/redox signalling in CO_2_/H^+^-sensitive brainstem pathways, as suggested by previous experimental studies and reviews concerning hyperbaric oxygen and oxidative stimuli [11,12]. However, SpO_2_, arterial blood gases, blood CO_2_ levels, ROS, and neural activity were not measured in this study. Therefore, these explanations remain speculative, cannot be directly verified from the present data, and pertain only to the phase pattern observed with the primary calculation.

### 4.3. Implications of Unchanged PETCO_2_

In the primary analysis, VE/VCO_2_ increased significantly during the steady phase, whereas no significant phase-related changes were observed in PETCO_2_. The increase in VE/VCO_2_ should not be interpreted as direct evidence of alveolar hyperventilation or CO_2_ washout because neither VE nor PETCO_2_ changed significantly. Rather, this finding indicates a modest, transient increase in the ratio of total ventilation to metabolic CO_2_ output. Because arterial CO_2_ tension, physiological dead space, and ventilation–perfusion matching were not assessed, the physiological mechanism underlying the transient increase in VE/VCO_2_ cannot be determined from the present data. It has been reported that PETCO_2_ may decrease during hyperoxia and that the associated cerebral vasoconstriction may influence the interpretation of cerebral blood flow responses [16]. In light of this, the fact that PETCO_2_ did not decrease significantly in this study suggests that mild HBO exposure at 1.3 ATA did not induce severe hypocapnic hyperventilation, at least not to the extent of causing a marked decrease in end-tidal CO_2_ pressure. Furthermore, because PETCO_2_ did not increase significantly, the response was also unlikely to involve marked CO_2_ retention. VE/VCO_2_ is not merely a simple ratio of ventilation to CO_2_ production but is a comprehensive indicator of ventilatory efficiency influenced by factors such as the ventilatory demand for CO_2_ elimination, the partial pressure of CO_2_ in arterial blood (PaCO_2_), dead-space ventilation, and the ventilatory–haemodynamic relationship [13]. Therefore, the increase in VE/VCO_2_ alone should not be interpreted as overt hyperventilation, particularly because PETCO_2_ remained stable. However, while PETCO_2_ is a useful non-invasive estimator of PaCO_2_ [17], it is not PaCO_2_ itself, and the relationship between the two is influenced by alveolar dead space and the PaCO_2_–PETCO_2_ difference. Furthermore, the concentration of inhaled oxygen has been reported to influence the PaCO_2_–PETCO_2_ difference; therefore, caution is required when treating PETCO_2_ as synonymous with PaCO_2_ under hyperoxic conditions [18].

Given these findings, the stability of PETCO_2_ indicates that no obvious end-tidal CO_2_ depletion or retention occurred; however, it does not directly indicate stable PaCO_2_ or blood CO_2_ content. Taken together, the primary analysis indicated a transient increase in VE/VCO_2_ during mild HBO exposure, without significant changes in VE or PETCO_2_; however, the sensitivity analysis showed that the direction of the VE/VCO_2_ change depended on the calculation method. From a respiratory biology perspective, the primary-analysis pattern may reflect a subtle interaction between hyperoxic exposure and CO_2_-related respiratory control rather than overt hyperventilation or a major disruption of CO_2_ homeostasis. Although these findings may be relevant when interpreting respiratory measurements during mild HBO exposure, their clinical significance remains unknown and should not be extrapolated to patient populations or medical HBOT settings because the study included only healthy young men and did not assess arterial blood gases or clinical outcomes.

### 4.4. Interpretation of Post-Phase Changes in VCO_2_ and VE/VCO_2_

During the post-phase, VCO_2_ decreased significantly compared with the pre- and steady phases, whereas VE and PETCO_2_ did not change significantly. VE/VCO_2_ decreased from the steady phase to the post-phase in the primary analysis but increased over the same comparison in the sensitivity analysis. Thus, the post-phase decrease in VCO_2_ was consistent, whereas the direction of the VE/VCO_2_ change depended on the calculation method. The decrease in VCO_2_ may reflect a post-exposure readjustment in metabolic CO_2_ output; however, the underlying mechanism remains uncertain. Metabolic indicators such as VO_2_, VCO_2_, and the respiratory quotient may undergo reversible changes following hyperoxic exposure [19].

However, the post-phase decrease in VCO_2_ should not be interpreted as direct evidence of metabolic suppression caused by HBO exposure. A previous human study conducted at a lower pressure than that used in the present protocol reported that 50 min of exposure to 1.25 ATA with 36% oxygen increased SpO_2_, blood flow, and resting energy expenditure following exposure [20]. However, the ventilatory and gas-exchange variables examined in the present study, including VE/VCO_2_, VE, VCO_2_, and PETCO_2_, were not assessed; therefore, it remains unclear whether the respiratory responses under this lower-pressure condition were consistent with or distinct from the transient VE/VCO_2_ increase observed with the primary calculation in the present study. Changes in autonomic nervous system activity following mild HBO exposure have also been demonstrated [7]. Therefore, the post-phase decrease in VCO_2_ and the calculation-dependent VE/VCO_2_ response may reflect a combination of factors, including the physiological recovery process following HBO exposure, the passage of time at rest, changes in breathing patterns, a state of relaxation, and changes in the measurement environment.

In conclusion, although the post-phase decrease in VCO_2_ may reflect a recovery-related change in metabolic CO_2_ output, the accompanying VE/VCO_2_ response was calculation-method-dependent, and the underlying mechanisms could not be determined from the measurements obtained in this study.

### 4.5. Interpretation of the Exploratory Condition × Phase Interaction Analyses and Supplementary NN Findings

Among the eight participants who completed both conditions, a significant Condition × Phase interaction was detected using both the primary and alternative VE/VCO_2_ calculation methods. This finding suggests that the phase-dependent pattern differed between the HBO and NN conditions. However, the within-condition phase effects and specific pairwise contrasts varied according to the VE/VCO_2_ calculation method; under the alternative calculation, the NN simple phase effect was significant, although no Bonferroni-adjusted NN pairwise comparison reached *p* < 0.05. Therefore, these interaction findings should be regarded as exploratory and do not supersede the primary HBO analysis conducted in all 16 participants. In the separate supplementary NN analysis using the primary VE/VCO_2_ calculation, no significant phase effects were observed for VE/VCO_2_, VCO_2_, or PETCO_2_, although VE showed a significant phase effect without significant pairwise differences. These exploratory findings suggest that elapsed time or repeated seated rest alone may not fully account for the observed phase patterns, but they do not establish a definitive between-condition effect. Because the NN condition included only eight participants and was not designed as a confirmatory, fully randomised, and counterbalanced between-condition comparison, these findings should be interpreted cautiously. The apparent difference in pre-phase VE/VCO_2_ between the HBO primary cohort and the NN supplementary subset may reflect the small sample size of the NN subset and separate-day variability in resting measurements; therefore, it should not be interpreted as a baseline imbalance between formal comparison groups.

### 4.6. Study Limitations and Future Research Directions

This study has some limitations. An important analytical limitation is that the direction of the VE/VCO_2_ phase change differed between the primary and alternative calculation methods, indicating that the primary directional finding was not robust to the order of averaging and ratio calculation. First, the participants were limited to healthy young men, and the NN condition included only eight participants as a supplementary reference condition. Therefore, caution is required when generalising the findings, and the present study cannot provide a definitive comparison between HBO and NN environments. Second, although an increase in ambient pressure was confirmed in this study, measurements of arterial blood gases, transcutaneous CO_2_, SpO_2_, blood CO_2_ levels, cerebral blood flow, and autonomic nervous activity were not obtained. Consequently, it was not possible to directly verify the extent to which the increase in inspired oxygen partial pressure was reflected in arterial blood oxygenation or whether the Haldane effect, central CO_2_/H^+^ sensitivity, autonomic regulation, or changes in metabolic rate contributed to the changes in VE/VCO_2_. In particular, while PETCO_2_ is a useful non-invasive indicator for estimating CO_2_ homeostasis, it does not directly measure PaCO_2_ or blood CO_2_ levels. Third, as the post-phase was conducted shortly after decompression, it was not possible to completely rule out the effects of room air exchange, the passage of time during rest, and measurement conditions. Fourth, as this study was conducted under a single condition of 1.3 ATA, the dose–response relationship with respect to atmospheric pressure, oxygen concentration, and exposure time remains unclear. Fifth, although measurements were conducted under seated resting conditions and participants were instructed to avoid strenuous physical activity on the day of testing, potential influences of circadian variation, meal timing, caffeine intake, sleep status, and psychological stress were not fully controlled and may have contributed to inter-individual variability in ventilatory and gas-exchange responses. Sixth, environmental humidity differed between the HBO and NN conditions. Although low humidity may affect airway comfort and mucociliary function, and inspired-gas humidification has shown limited effects on pulmonary function under medical HBO conditions [21,22], its influence on resting ventilatory and gas-exchange variables under mild HBO conditions remains unclear. Therefore, humidity cannot be excluded as a potential confounding factor.

Future studies should prespecify the VE/VCO_2_ calculation method, evaluate the robustness of findings across alternative calculation approaches, and use a fully powered, randomised, and counterbalanced crossover design in which all participants complete both the HBO and NN conditions. Furthermore, evaluations of arterial blood gases, transcutaneous CO_2_, SpO_2_, blood CO_2_ levels, cerebral blood flow, and autonomic nervous activity would aid in investigating the mechanisms underlying changes in VE/VCO_2_ during mild HBO exposure and the dose–response characteristics of VE/VCO_2_.

## 5. Conclusions

Using the primary calculation, mild HBO exposure at 1.3 ATA was associated with a modest, transient increase in VE/VCO_2_ in healthy young men during seated rest, without significant changes in VE or PETCO_2_. However, the alternative calculation produced the opposite phase-dependent pattern, indicating that the direction of the VE/VCO_2_ change depended on the calculation method. Accordingly, these findings should not be interpreted as evidence of alveolar hyperventilation, CO_2_ washout, or a definitive alteration in respiratory regulation. The Haldane effect and central respiratory regulation remain plausible but unverified explanations for the pattern observed with the primary calculation. Fully powered, randomised, and counterbalanced crossover studies with prespecified VE/VCO_2_ calculation methods and direct physiological measurements are required.

## Figures and Tables

**Table 1:** Participant characteristics (*n* = 16).

Variable	Mean ± SD
Age (years)	22.1 ± 3.1
Height (cm)	173.5 ± 6.9
Weight (kg)	73.5 ± 10.8
BMI (kg·m^−2^)	24.3 ± 3.0
Fat mass (kg)	13.1 ± 6.5
Body fat (%)	17.3 ± 6.3
Total body water (L)	44.2 ± 5.4
BMR (kcal·day^−1^)	1674 ± 159

Values are presented as mean ± standard deviation (SD). BMI, body mass index; BMR, basal metabolic rate; SD, standard deviation.

**Table 2:** Physiological responses during the 20–50 min period after the absolute pressure reached 1.3 ATA (*n* = 16).

Variable	Mean ± SD	95% CI (Low)	95% CI (High)
VE (L·min^−1^)	11.9 ± 1.8	10.9	12.8
VCO_2_ (mL·min^−1^)	298.8 ± 49.9	272.2	325.4
VE/VCO_2_	43.8 ± 4.1	41.6	46.0
PETCO_2_ (mmHg)	34.9 ± 2.8	33.4	36.4

Values are presented as mean ± standard deviation (SD) with 95% confidence intervals (CIs). For the primary calculation, VE/VCO_2_ was calculated for each 30 s bin by dividing bin-averaged VE by bin-averaged VCO_2_ after both variables had been expressed in consistent volume units. The resulting bin-wise ratios were then averaged over the 20–50 min steady-state period for each participant. VE/VCO_2_, ventilatory equivalent for carbon dioxide; VE, minute ventilation; VCO_2_, carbon dioxide output; PETCO_2_, partial pressure of end-tidal carbon dioxide; BTPS, body temperature and pressure, saturated. VE/VCO_2_ is dimensionless; VE is expressed in L·min^−1^ (BTPS), VCO_2_ in mL·min^−1^ (BTPS), and PETCO_2_ in mmHg.

**Table 3:** Linear mixed model analysis of phase-dependent changes in ventilatory and gas-exchange variables (*n* = 16). (**A**) Model estimates and omnibus phase effects. (**B**) Pairwise comparisons and effect sizes.

(**A**)						
**Variable**	**Phase**	**EMM ± SE**	**95% CI (Low)**	**95% CI (High)**	***F***	***p***
VE/VCO_2_	Pre	39.9 ± 1.0	37.8	42.0	16.984	<0.001
	Steady	43.8 ± 1.0	41.7	45.9		
	Post	38.8 ± 1.0	36.7	40.9		
VE (L·min^−1^)	Pre	12.2 ± 0.5	11.2	13.1	2.426	0.105
	Steady	11.8 ± 0.5	10.9	12.8		
	Post	11.4 ± 0.5	10.4	12.3		
VCO_2_ (mL·min^−1^)	Pre	302.0 ± 13.1	274.6	329.3	8.009	0.002
	Steady	298.8 ± 13.1	271.5	326.1		
	Post	274.6 ± 13.1	247.3	301.9		
PETCO_2_ (mmHg)	Pre	35.1 ± 0.8	33.4	36.8	1.371	0.281
	Steady	34.9 ± 0.8	33.2	36.6		
	Post	35.8 ± 0.8	34.2	37.5		
(**B**)						
**Variable**	**Bonferroni-Adjusted *p***			**Hedges’ *g_av_***		
	**Pre–Steady**	**Steady–Post**	**Pre–Post**	**Pre–Steady**	**Steady–Post**	**Pre–Post**
VE/VCO_2_	<0.001	<0.001	0.455	−0.966	1.229	0.263
VE (L·min^−1^)	1.000	0.997	0.122	0.179	0.259	0.438
VCO_2_ (mL·min^−1^)	1.000	0.009	0.020	0.061	0.462	0.523
PETCO_2_ (mmHg)	1.000	0.483	0.449	0.057	−0.292	−0.235

Values are presented as estimated marginal means (EMM) ± standard error (SE) with 95% confidence intervals (CI). For VE/VCO_2_, values were based on the primary calculation, in which bin-wise ratios were averaged within each phase. Phase effects were evaluated using linear mixed models with restricted maximum likelihood estimation. Pairwise comparisons were adjusted using the Bonferroni method. Effect sizes are reported as Hedges’ *g*_av_, calculated as the first-named phase minus the second-named phase in each comparison. VE/VCO_2_, ventilatory equivalent for carbon dioxide; VE, minute ventilation; VCO_2_, carbon dioxide output; PETCO_2_, partial pressure of end-tidal carbon dioxide; BTPS, body temperature and pressure, saturated. VE/VCO_2_ is dimensionless; VE is expressed in L·min^−1^ (BTPS); VCO_2_ in mL·min^−1^ (BTPS); and PETCO_2_ in mmHg.

## Data Availability

The data sets used and analysed during the current study are available from the corresponding author on reasonable request.

## Supplementary Materials

The following supporting information can be downloaded at: https://www.mdpi.com/article/10.3390/biology15151254/s1, Table S1: Supplementary steady-state exposure characteristics and physiological responses during the 20–50 min period at 1.3 ATA (*n* = 16); Table S2: Linear mixed model analysis of phase-dependent changes in ventilatory and gas-exchange variables under normobaric normoxia (*n* = 8). Table S3: Sensitivity analysis of phase-dependent changes in VE/VCO_2_ calculated as phase-averaged VE divided by phase-averaged VCO_2_ under the mild hyperbaric oxygen condition (*n* = 16); Table S4: Exploratory Condition × Phase interaction analyses of VE/VCO_2_ using two calculation methods in participants who completed both the mild hyperbaric oxygen and normobaric normoxic conditions (*n* = 8).

## Abbreviations

The following abbreviations are used in this manuscript:

ATA	Atmospheres absolute
BMI	Body mass index
CO_2_	Carbon dioxide
HBO	Hyperbaric oxygen
HBOT	Hyperbaric oxygen therapy
NN	Normobaric normoxia
PETCO_2_	Partial pressure of end-tidal carbon dioxide
SpO_2_	Peripheral oxygen saturation
VE	Minute ventilation
VCO_2_	Carbon dioxide output
VO_2_	Oxygen uptake
VE/VCO_2_	Ventilatory equivalent for carbon dioxide
VE/VO_2_	Ventilatory equivalent for oxygen
