# Supplementary material for: Mild Hyperbaric Oxygen Exposure Is Associated with Calculation-Method-Dependent Changes in VE/VCO2 During Seated Rest
Source: Biology (Basel). 2026 Jul 30;15(15):1254. doi: 10.3390/biology15151254 (PMC13463848; doi:10.3390/biology15151254)
Supplement: Supplementary file 1 [file biology-15-01254-s001.zip › biology-4416968-supplementary.pdf]

Supplementary Materials

Table S1. Supplementary steady-state exposure characteristics and physiological responses during the 20–50 min period at 1.3 ATA (n = 16).

Variable	Mean ± SD			95% CI (low)	95% CI (high)
Barom (mmHg)	987.5	±	3.6	985.7	989.4
RR (breaths·min^{-1})	17.9	±	3.2	16.2	19.6
TVE (L)	0.77	±	0.22	0.66	0.89
VO_2 (mL·min^{-1})	349.2	±	59.6	317.4	380.9
R	0.86	±	0.05	0.83	0.89
VE/VO_2	38.9	±	3.9	36.8	41.0
$PETO_2$ (mmHg)	179.1	±	5.0	176.4	181.8
HR (beats·min^{-1})	68.5	±	7.9	64.3	72.7

Values are presented as mean ± standard deviation (SD) with 95% confidence intervals (CI). Barom, barometric pressure; RR, respiratory rate; TVE, tidal volume; VO_2, oxygen uptake; R, respiratory exchange ratio; VE/VO_2, ventilatory equivalent for oxygen; $PETO_2$, partial pressure of end-tidal oxygen; HR, heart rate; BTPS, body temperature and pressure, saturated. R and VE/VO_2 are dimensionless; VO_2 is expressed in mL·min^{-1} (BTPS), and $PETO_2$ in mmHg.

Table S2. Linear mixed model analysis of phase-dependent changes in ventilatory and gas-exchange variables under normobaric normoxia (n = 8).

Variable	Phase	EMM ± SE			95% CI (low)	95% CI (high)	F	p	Pre–Steady p	Steady–Post p	Pre–Post p
VE/VCO_2	Pre	35.8	±	1.8	31.8	39.7	1.941	0.181	0.873	1.000	0.297
	Steady	34.3	±	1.8	30.4	38.3					
	Post	33.4	±	1.8	29.5	37.4					
VE (L·min^{-1})	Pre	12.1	±	0.9	10.2	14.0	5.606	0.016	0.115	1.000	0.055
	Steady	10.8	±	0.9	8.9	12.8					
	Post	10.6	±	0.9	8.7	12.6					
VCO_2 (mL·min^{-1})	Pre	290.6	±	15.3	257.2	324.0	1.061	0.373	0.645	1.000	1.000
	Steady	275.9	±	15.3	242.5	309.3					
	Post	281.2	±	15.3	247.8	314.6					
$PETCO_2$ (mmHg)	Pre	37.3	±	1.7	33.5	41.0	1.937	0.183	0.522	1.000	0.398
	Steady	39.5	±	1.7	35.7	43.2					
	Post	39.7	±	1.7	36.0	43.5					

Values are presented as estimated marginal means (EMM) ± standard error (SE) with 95% confidence intervals (CI). Phase effects were evaluated using linear mixed models with restricted maximum likelihood estimation. Pairwise comparisons were adjusted using the Bonferroni method, and all pairwise comparisons among phases are reported. VE/VCO_2, ventilatory equivalent for carbon dioxide; VE, minute ventilation; VCO_2, carbon dioxide output; $PETCO_2$, partial pressure of end-tidal carbon dioxide; BTPS, body temperature and pressure, saturated. VE/VCO_2 is dimensionless; VE is expressed in L·min^{-1} (BTPS); VCO_2 in mL·min^{-1} (BTPS); and $PETCO_2$ in mmHg.

Table S3. Sensitivity analysis of phase-dependent changes in VE/VCO_2 calculated as phase-averaged VE divided by phase-averaged VCO_2 under the mild hyperbaric oxygen condition (n = 16).

Panel A. Estimated marginal means by phase.

Phase	EMM ± SE			95% CI (low)	95% CI (high)
Pre	42.5	±	1.2	40.0	45.0
Steady state	39.9	±	1.2	37.4	42.4
Post	43.2	±	1.2	40.7	45.7

Panel B. Omnibus phase effect.

df	F	p
2, 28.172	12.313	<0.001

Panel C. Bonferroni-adjusted pairwise comparisons.

Phase	Mean difference	95% CI (low)	95% CI (high)	Adjusted p	Hedges' g_{av}
Pre–Steady state	2.5	0.5	4.6	0.010	0.618
Steady state–Post	−3.2	−5.3	−1.2	<0.001	−0.752
Pre–Post	−0.7	−3.4	2.0	1.000	−0.134

Values are presented as estimated marginal means (EMM) ± standard error (SE) with 95% confidence intervals (CI). VE/VCO_2 was calculated for each participant as phase-averaged minute ventilation (VE) divided by phase-averaged carbon dioxide output (VCO_2), with VCO_2 converted from $mL \cdot min^{-1}$ to $L \cdot min^{-1}$ before calculation. Phase effects were evaluated using a linear mixed model with restricted maximum likelihood estimation and a first-order autoregressive covariance structure. Pairwise comparisons were Bonferroni-adjusted. VE/VCO_2 is dimensionless.

Table S4. Exploratory Condition × Phase interaction analyses of VE/VCO_2 using two calculation methods in participants who completed both the mild hyperbaric oxygen and normobaric normoxic conditions (n = 8).

Panel A. Tests of fixed effects.

Calculation method	Effect	df	F	p
Mean of bin-wise ratios	Condition	1, 6.677	14.754	0.007
	Phase	2, 26.255	9.578	<0.001
	Condition × Phase	2, 26.255	10.577	<0.001
Ratio of phase means	Condition	1, 7.419	2.790	0.136
	Phase	2, 26.487	3.278	0.053
	Condition × Phase	2, 26.487	5.576	0.010

Panel B. Estimated marginal means and simple phase effects.

Calculation method	Condition	Pre	Steady	Post	Simple phase effect
Mean of bin-wise ratios	HBO	39.8 ± 1.7[a]	44.6 ± 1.8[b]	38.8 ± 1.7[a]	$F(2, 25.891) = 22.126$, $p < 0.001$
	NN	35.8 ± 1.5[a]	34.3 ± 1.5[a]	33.4 ± 1.5[a]	$F(2, 25.891) = 1.474$, $p = 0.248$
Ratio of phase means	HBO	41.9 ± 1.9[ab]	40.6 ± 1.9[a]	44.7 ± 1.9[b]	$F(2, 26.144) = 6.826$, $p = 0.004$
	NN	41.5 ± 1.8[a]	39.1 ± 1.8[a]	37.8 ± 1.8[a]	$F(2, 26.144) = 3.404$, $p = 0.049$

Values are estimated marginal means (EMM) ± standard error (SE). Within each row, values without a common superscript letter differ significantly after Bonferroni adjustment ($p < 0.05$). Mean of bin-wise ratios and ratio of phase means represent the phase average of bin-wise VE/VCO_2 and phase-averaged VE divided by phase-averaged VCO_2, respectively. HBO, mild hyperbaric oxygen; NN, normobaric normoxia.
